# Letter to Editor: Carpal tunnel syndrome due to an atypical deep soft tissue leiomyoma: The risk of misdiagnosis and mismanagement

**DOI:** 10.1186/1477-7819-6-22

**Published:** 2008-02-20

**Authors:** Giuseppe Granata, Carlo Martinoli, Costanza Pazzaglia, Pietro Caliandro, Luca Padua, Diana Ferraro

**Affiliations:** 1 Institute of Neurology, Università Cattolica del Sacro Cuore, Largo F. Vito 1, 00168 Rome, Italy; 2 Fondazione Don Carlo Gnocchi, Rome, Italy; 3 Cattedra di Radiologia "R", DICMI, Università di Genova, Italy

## Abstract

A response to Chalidis et al: Carpal tunnel syndrome due to an atypical deep soft tissue leiomyoma: The risk of misdiagnosis and mismanagement. *World J Surg Oncol *2007, 5:92.

## 

We read with great interest the article by Chalidis *et al*. [1], on the risk of misdiagnosis and mismanagement of carpal tunnel syndrome due to an atypical deep soft-tissue leiomioma. The authors report a case of a 32 year-old man with symptoms that were attributed to carpal tunnel syndrome (CTS), confirmed by a nerve conduction study, which did not improve after surgery. Magnetic resonance imaging (MRI) was performed and it showed a deep soft-tissue mass located on the palm of the hand, compatible with leiomyoma. In the discussion, the authors underline the importance, especially in young people, to hypothesize the presence of an underlying tumour when residual symptoms persist after initial surgical treatment.

MRI is known to be a good technique to diagnose nerve or deep soft tissue tumors. Nevertheless, with the introduction of broadband high-frequency transducers, nerve ultrasound (US) is a rapidly expanding technique because it is able to directly visualize nerve abnormalities, provide precise information on surrounding tissues and, in case of CTS, show whether median nerve compression is due to a tumour or whether it is idiopathic [2].

Usually we diagnose CTS on the basis of the clinical picture and of a neurophysiological evaluation. We use neuroimaging exams in case of atypical neurophysiological findings, atypical clinical symptoms, dissociation between neurophysiological and clinical findings or, as in the case reported by Chalidis [1], when there is not benefit after surgical treatment. In a previous paper [3] we reported five cases of median nerve schwannoma, which clinically simulated a carpal tunnel syndrome and we demonstrated that it is important to examine the median nerve, not only at the wrist, but also out of the wrist.

Both MRI and US allow us to visualize nerve or soft-tissue tumors and they allow us to distinguish between tumors originating from the nerve or from soft tissues. Although it is often impossible to surely differentiate between schwannoma (figure 1) and neurofibroma, which are the most frequent nerve tumors, some US features may distinguish between the two [4]. In our experience, MRI always confirmed ultrasonography findings and did not provide any further useful information for the surgeons.

**Figure 1:**
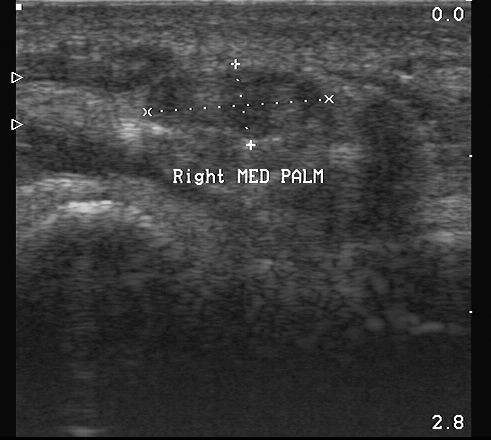
Schwannoma of median nerve at palm: A case of Schwannoma: the picture shows an increased cross sectional area of median nerve at palm.

In conclusion, we agree with Chalidis [1] that it is important to add neuroimaging examinations to clinical and neurophysiological assessments in atypical CTS. However, we think that, being US an inexpensive and easily available method which also provides a dynamic examination, it may be the first-line approach to the nerve. The cost-benefit ratio is in favour of using US rather than MRI for a number of reasons: 1) US is less time consuming; it only takes around 5 minutes [5] to carry out an US evaluation of a wrist, while a wrist MRI examination takes around 25 minutes; the MRI may last up to 35 minutes if it is carried out with contrast medium (CM); 2) US is less expansive; in our hospital, the price of a musculoskeletal US is 63 euros (about 92 U.S. dollars), while the price of a MRI of the same district is 344 euros (about 504 U.S. dollars) without CM and 527 euros (about 772 U.S. dollars) with CM (data supplied by the national sanitary system). We think that MRI may be useful in cases in which US gives negative results, but a clinical suspect of tumour persists, or when the tumour is localized in a deep portion of the nerve, which is not easily visualized with US, especially in obese people.

Finally, we want to highlight that US and MRI can also be very useful to visualize nerve or soft-tissue tumors in districts other from the hand [6].

## Abbreviations

Carpal Tunnel Syndrome (CTS); Magnetic Resonance Imaging (MRI); Nerve Ultrasound (US); Contrast Medium (CM).

## Competing interests

The author(s) declare that they have no competing interests.

## Authors' contributions

GGliterature review and preparation of draft manuscript. MC**, **PC, and CPhelped in preparation of manuscript. LPhelped in preparing the draft manuscript and edited the final version.
